# Thermoresponsive Hydrogel Containing *Viscum album* Extract for Topic and Transdermal Use: Development, Stability and Cytotoxicity Activity

**DOI:** 10.3390/pharmaceutics14010037

**Published:** 2021-12-24

**Authors:** João V. D. C. Batista, Ana Paula S. Matos, Adriana P. Oliveria, Eduardo Ricci Júnior, Zaida M. Freitas, Catarina A. Oliveira, Helena K. Toma, Marcia A. M. Capella, Leandro M. Rocha, Ulrike Weissenstein, Stephan Baumgartner, Carla Holandino

**Affiliations:** 1 Laboratório Multidisciplinar em Ciências Farmacêuticas, Faculdade de Farmácia, Universidade Federal do Rio de Janeiro, Rio de Janeiro 21941-902, RJ, Brazil; j.dacostabatista@vfk.ch (J.V.D.C.B.); passosoliv@hotmail.com (A.P.O.); 2 Hiscia Institute, Society for Cancer Research, 4144 Arlesheim, Switzerland; u.weissenstein@vfk.ch; 3 Laboratório de Desenvolvimento Galênico, Faculdade de Farmácia, Universidade Federal do Rio de Janeiro, Rio de Janeiro 21941-902, RJ, Brazil; anapaulasmatos@gmail.com (A.P.S.M.); ricci@pharma.ufrj.br (E.R.J.); zaidafarmacia@gmail.com (Z.M.F.); 4 Laboratório de Alimentos, Instituto Federal de Educacão, Ciência e Tecnologia do Rio de Janeiro, Rio de Janeiro 20270-021, RJ, Brazil; catarina.oliveira@ifrj.edu.br; 5 Laboratório de Diagnóstico Molecular e Hematologia, Faculdade de Farmácia, Universidade Federal do Rio de Janeiro, Rio de Janeiro 21941-902, RJ, Brazil; hktoma@globo.com; 6 LaRBio Carlos Chagas Filho, Biophysics Institute, Universidade Federal do Rio de Janeiro, Rio de Janeiro 21941-902, RJ, Brazil; mamcapella@gmail.com; 7 Laboratório de Tecnologia de Produtos Naturais, Departamento de Tecnologia Farmacêutica, Universidade Federal Fluminense, Niteroi 24241-000, RJ, Brazil; lean.machado@gmail.com; 8 Institute of Complementary and Integrative Medicine, University of Bern, 3012 Bern, Switzerland; 9 Institute for Integrative Medicine, University of Witten/Herdecke, 58455 Herdecke, Germany

**Keywords:** mistletoe, *Viscum album*, thermoresponsive hydrogel, rheology, stability, toxicity test, permeation

## Abstract

*Viscum album* L. (*Santalaceae*), also known as European mistletoe, is a semi-parasitic plant that grows on different host trees. Our group recently demonstrated the antitumoral activity of ethanolic *V. album* extracts in vitro, depending on the dose and the host tree, *V. album* ssp *abietis* from *Abies alba* being the most active extract. The goal of this work focused on the development of a new topical formulation containing *V. album* extracts, evaluation of in vitro toxicity and ex vivo skin permeation assays. The Poloxamer 407 hydrogel containing 5% of dry (VA_DEH) or aqueous (VA_AEH) extract presented dermal compatible pH and microbiological stability for 180 days. The hydrogels flow curve presented a non-linear relation, characteristic of non-Newtonian fluids, and the mean viscosity for the VA_DEH and VA_AEH was 372.5 ± 7.78 and 331.0 ± 2.83 Pa.s, respectively, being statistically different (Welch’s *t* test; *p* < 0.01). Additionally, WST-1 in vitro assays revealed a dose-dependent toxicity for both formulations and VA_DEH presented a higher activity than the VA_AEH. The promising cytotoxic potential of VA_DEH lead to the ex vivo skin permeation assay with 2.73 ± 0.19 µg/cm^2^ of chlorogenic acid, which permeated at 8 h, showing a transdermal potential. These in vitro results support the idea that VA_DEH is a novel promising candidate for mistletoe therapy. Therefore, further in vivo and pre-clinical experiments should be performed to evaluate the safety and efficacy of this new dermic delivery system.

## 1. Introduction

The high incidence of cancer is a very concerning subject in public health, especially its effect on quality of life and longevity. Research with natural therapeutic products in cancer treatment have revealed their importance and are contributing as anti-cancer and immunomodulatory sources [1,2]. From 1940 and 2014, approximately 55% of antitumoral drugs approved for cancer treatment came from natural resources [3]. As a prominent example, *Viscum album* L. (white-berry or European mistletoe) is widely studied [4,5], since it presents a great variety of bioactive compounds [5] and an irrefutable therapeutic potential [6].

Aqueous extracts of *Viscum album* (VA) have a long history in complementary therapy against cancer, primarily in Central Europe [7,8]. The most studied active substances in aqueous extracts of VA are lectins and viscotoxins [9]. These compounds show immunomodulatory activities and are able to induce cell death by apoptosis and necrosis mechanisms [10,11,12]. In addition, using a comprehensive metabolome analysis, more than 200 primary and secondary metabolites were putatively annotated when fermented aqueous extracts of VA from *Malus domestica* and *Pinus sylvestris* were analyzed [13].

The phytochemical composition of the extracts from VA is related to their antitumoral potential. These extracts also present a host tree dependence, which was confirmed when methanolic extracts were analyzed. Furthermore, the untargeted metabolomic approach led us to identify host tree specific VA biomarkers, which might be important for the herbal quality control parameters [14].

The influence of the host tree on the cytotoxic potential of mistletoe is an exciting research topic that has been previously highlighted when five different ethanolic extracts prepared from the three European subspecies (*album*, *austriacum*, and *abietis*) were evaluated by an in vitro model [15]. These studies emphasize the antitumoral potential of ethanolic soluble substances from VA for cancer pharmacotherapy.

The development of a transdermal delivery system containing hydrophilic and hydrophobic components of VA extracts can be considered as an additional local approach for antitumoral therapy. The use of hydrogels has become more popular because of its high content of water, soft consistency, flexibility, and biocompatibility [16,17,18].

In addition, thermoresponsive hydrogel exhibit liquid characteristics at low temperatures and becomes viscous at higher temperatures, such as at body temperature, which facilitates administration and accessibility when applied in drug delivery systems [17,19,20,21]. Furthermore, the hydrogel thermosensitivity might help to increase the in vivo lifetime and the contact with the tissue. All this gives a wide possibility for optimizing cancer treatments such as localized and targeted drug delivery systems with easier accessibility [17,18,22,23].

Poloxamers are non-ionic, non-toxic, water soluble triblock copolymers family composed of poly-(ethylene oxide) (PEO) and hydrophobic poly-(propylene oxide) (PPO) [21,24,25,26]. These polymers have some advantages, like commercial availability, wide range of molecular weights, and PEO/PPO composition [21,24]. Moreover, some poloxamers like Poloxamer 407 and Poloxamer 188 have been approved for pharmaceutical formulations by the FDA. Poloxamer 407 formulations can be used for drug, small molecules, peptides, biological molecules deliveries, and especially for controlled release [25].

The present work focused on the development of a new pharmaceutical formulation for the transdermal delivery of VA extracts as a new route of administration with topical and systemic effect. Besides the advantages concerning the use of poloxamer thermosensitivity hydrogel, its use for VA therapy could avoid the VA injection’s invasiveness by a suitable dermic delivery administration. Stability studies were carried out to verify chemical and rheological parameters. Additionally, in vitro cytotoxicity assays were performed using tumoral and non-tumoral cell lines, showing the promising therapeutic potential of the VA dry extract conjugated to a thermosensitive hydrogel.

## 2. Materials and Methods

### 2.1. Plant Harvesting and Identification

The ripe berries, leaves, and stems of European VA ssp. *abietis* from *Abies alba* were harvested in January of 2019 in their natural habitat (Nuglar-St Pantaleon, Solothurn canton, Switzerland). The climate at the site is temperate classified as Cfb by the Köppen–Geiger system, with annual average rainfall of 1413 mm and average temperature of 0.6 °C/33.1 °F [27]. The plant was identified by Dr Marcelo Guerra Santes (Universidade Estadual do Rio de Janeiro). Voucher specimen (C.H. Quaresma 18.328) was deposited at the Herbarium of the Faculdade de Formação de Professores, Universidade Estadual do Rio de Janeiro, Brazil, as described before [15].

### 2.2. Viscum album Ethanolic Extract

All solvents and reagents exhibited analytical purity quality. The fresh material (5 g) was fragmented into segments smaller than 5 cm, then dried in an oven at 105 °C for 2 h, as described before [15], following the Brazilian Homeopathic Pharmacopeia [28] and the ANSM French Pharmacopeia [29]. After the solid residue percentage calculation, the total volume of the ethanolic extract as well as the volume and the concentration of ethanol used in the maceration process were determined. Then, the maceration extractive process was conducted at room temperature in ethanol 70% *w*/*w*. To increase the efficiency of the extraction process, the VA ethanolic solution was shaken by hand for 60 s, twice a day. After 3 weeks, the macerate was filtered and kept at 20 ± 5 °C. The ethanolic extract final concentration was 47% *v*/*v* [29].

### 2.3. Viscum album Ethanolic Dry Extract

The ethanolic extract was transferred to a balloon and the solvent was evaporated at 40 °C (Water Bath, Büchi, Flawil, Switzerland) under vacuum (Vacuum Pump V-700, Büchi, Flawil, Switzerland). Lastly, the residue was frozen at −80 °C and subsequently lyophilized (Christ Beta 2-8 LD) for a period of 24 h (−43 °C/0.09 mbar), obtaining the ethanolic dry extract of VA ssp. *abietis* (VA_DE).

#### 2.3.1. Thin Layer Chromatography

VA_DE was analyzed by thin layer chromatography (TLC) following French Pharmacopeia [29]. Briefly, samples (10–20 µL/band) were applied as 10 mm bands with 10 mm track distance onto the plate by a capillary at 10 mm distance from the lower edge of the plate. TLC separations were carried out in a chamber (210 × 100 mm) saturated with distilled water/methanol/glacial acetic acid/dichloromethane, 2/3/8/15 (*v*/*v*/*v*/*v*) as mobile phase. These bands were visualized under UV light (365 nm) before and after the use of revealing spray solution containing diphenylboriloxyethilamine/polyetileneglicol (NP/PEG).

#### 2.3.2. Determination of VA_DE Total Flavonoid Content

The flavonoid concentration of ethanolic extract was determined spectrophotometrically in the UV region (360 nm), in comparison with the rutin standard curve, adapted from Rolim et al. [30]. The following concentration range was used: 5.0, 10.0, 15.0, 20.0, 25.0, and 30.0 µg/mL. The absorption values were plotted to provide a linear rutin calibration curve with r^2^ > 0.99. A mixture of ethanol 95% and acetic acid 0.02 M (99:1) was used as the solvent in all solution preparations. The readings were performed in duplicate, as previously described [15].

#### 2.3.3. UHPLC-DAD-MS/MS

The VA_DE analysis was conducted in an ultrahigh liquid performance liquid chromatography (UHPLC) Dionex Ultimate 3000, coupled with diode array detector (DAD) (Thermo Fisher Scientific, Waltham, MA, USA) and tandem mass spectrometry (MS) with electrospray ionization (LCQ Fleet Ion Trap-Thermo Fisher Scientific, Waltham, MA, USA) in a reverse phase column (C-18, 250 mm × 4.6 mm × 5.0 µm; Kromasil, Akzo Nobel, Nashville, TN, USA). The sample was prepared as follows: a solution of acidified acetonitrile and acidified water with 0.1% *v*/*v* of formic acid each was prepared in a proportion of 1:9 (*v*/*v*) (solution T), respectively. Then, 50 mg of the VA_DE was solubilized in 1 mL of ethanol 45% *v*/*v* and added in a 5 mL volumetric balloon, filling it up with solution T. At the end, the sample was centrifuged (6400 rpm, 10 min) and filtered in a 0.45 µm membrane filter.

Separation was performed in a reverse phase column (C-18, 250 mm × 4.6 mm × 5.0 µm; Kromasil, Akzo Nobel, Nashville, TN, USA) using water-formic acid 0.1% *v*/*v* (A) and acetonitrile formic acid 0.1% *v*/*v* (B) as mobile phases: (i) 0–20 min, 10% B, (ii) 20–25 min, 10–15% B, (iii) 25–45 min, 15% B, (iv) 45–50 min, 15–100% B, (v) 50–55 min 100% B, (vi) 55–57 min 100–10%, and (vii) 57–65 min 10% B, as described in the monograph of VA [29]. The flow rate used was 1.0 mL/min and the injected volume of 70 µL. The absorption spectrum in the UV-Vis was obtained at a range from 200 to 400 nm. Mass spectra were performed in negative ion mode [29,31].

#### 2.3.4. HPLC-UV-Vis Analysis

The VA_DE was analyzed as described in VA monograph with modifications [29]. For this purpose, a high-performance liquid chromatography (HPLC) Dionex Ultimate 3000 equipped with ultraviolet detection (Thermo Fisher Scientific, Waltham, MA, USA) was used in a reverse phase column (C-18, 250 mm × 4.6 mm × 5.0 µm; Kromasil, Akzo Nobel, Nashville, TN, USA). The sample was prepared as described on (Section 2.3.3) [29,31].

The flow rate used was 1.0 mL/min and the injected volume of 100 µL. The chromatographic peaks were determined at 325 nm.

### 2.4. Viscum album Aqueous Extract

The aqueous extract of VA (VA_AE), trade name Iscador^®^ (batch 1804/8141), was donated by the pharmaceutical company Iscador AG (Arlesheim, Switzerland). Following methodology previous validated [32], the viscotoxin content was used as a chemical parameter, in which 200 mg of fresh plant/mL of aqueous VA ssp *abietis* extract presented 421 µg/mL of total viscotoxin.

### 2.5. Viscum album Thermoresponsive Hydrogels

The thermoresponsive hydrogels were prepared with 20% *w*/*w* of Poloxamer 407 dispersed in distilled water and were kept under refrigeration for at least 24 h. Then 5% *w*/*w* of propylene glycol, 5% *w*/*w* of transcutol^®^ (diethylene glycol monoethyl ether), and 5% *w*/*w* of the VA_DE was solubilized under magnetic stirrer, at 4 °C using an ice bath, resulting in VA_DE hydrogel (VA_DEH). The VA hydrogel containing 5% *w*/*w* of the VA_AE was prepared with the same ingredients and proportions (Table 1), following the same procedure, resulting in VA_AE hydrogel (VA_AEH). All reagents used in the hydrogel’s preparation were obtained from Sigma-Aldrich (Buchs, Switzerland).

#### 2.5.1. Rheology

Thermal bath Tecnal TE 2015 for temperature control and Rheometer Anton Paar MCR 302 with Peltier system were used for rheological measurements. Results were obtained by Rheoplus/32 V.3.61 software. The rotational and oscillatory analyses were carried out using a cone-and-plate (CP) geometry (40 mm diameter and angle of 2°) at 32 °C. A temperature sweep between 50 to −10 °C was carried out to determine the viscosity as a function of temperature. Hydrogels’ viscosity curve was obtained applying a shear rate in the range from 1 to 100 s^−1^.

Three-interval thixotropic test (3ITT) was carried out to evaluate the thixotropy by means of percentage values of structure recovery for each sample. First, a low shear rate of 1 s^−1^ was applied and then a high shear rate of 100 s^−1^ was applied. Lastly, the recovery was determined after 10 and 60 s at the low shear rate of 1 s^−1^. Amplitude sweeps (constant frequency of 10 rad.s^−1^) and frequency (amplitude of 0.1%) tests were also carried out to obtain the structural characteristics of the samples, like the flow point and strength of the hydrogels.

#### 2.5.2. Stability Studies

The hydrogels were stored in a polypropylene airless dispenser (E. Anwander & Cie AG, Oberwill, Switzerland) with volume up to 15 mL. The airless dispenser system consisted of two mains parts: a container with piston and a dispenser head with fitted cap. This airless dispenser system avoided contact with ambient air and consequently oxidation reactions. For the stability studies, three different batches of each hydrogel were prepared and stored in a climatic chamber for accelerated stability evaluation, with controlled temperature (40 ± 5 °C) and humidity (75 ± 5%), according to the Brazilian Pharmacopeia [33]. The following assays were done at 7, 15, 30, 60, 90, and 180 days after manufacturing: pH (Bante Instrument Model 922), viscosity (Rheometer Anton Paar MCR-302), and microbiological analyses.

### 2.6. In Vitro Assays

#### 2.6.1. Cell Lines and Culture Conditions

Yoshida mouse sarcoma (ATCC, Rockville, MD, USA), Molt-4 human lymphoblastic leukemia (ATCC, Rockville, MD, USA), and non-tumor HaCat human adult keratinocytes (Institute of Pathology, University Hospital Basel, Basel, Switzerland) were cultured in RPMI-1640 medium, supplemented with 5% heat-inactivated fetal calf serum (FCS), 2 mM L-glutamine, 1% penicillin—streptomycin in a humidified atmosphere with 5% CO_2_ at 37 °C. Cell lines were maintained in exponential growth and cells from subconfluent monolayers (Yoshida and HaCat) were collected using trypsin-EDTA to carry out the experiments.

#### 2.6.2. Cell Viability Assay

Cell viability was evaluated by the WST-1 colorimetric methodology. Briefly, 90 µL of each cellular suspension containing 5 × 104 cells/mL were precultured in 96-well plates. After 24 h, 90 µL of hydrogels previously weighed and solubilized in cellular culture medium were added in concentrations varying from 2 to 200 mg/mL. Cellular viability rates were measured after 24 h of incubation by the addition of 20 µL of WST-1 to each well, incubated for 1:30 h. Absorption at 450 nm and 650 nm were measured in a multi-well plate reader (Labsystems Multscan RC, Helsinki, Finland). Cells without any treatment were used as background control, and cells treated with hydrogel without VA were used as vehicle control. Their effect on the cell viability was also evaluated. The percentage of viable cells was calculated in relation to vehicle control cells, using mean values from at least three independent experiments that were performed in triplicates. The IC50 was calculated using GraphPad 8 Software.

### 2.7. VA_DEH Ex Vivo Permeation Study

#### 2.7.1. Skin Permeation Method

The ex vivo skin permeation study of VA_DEH was carried out in a Franz type vertical cell diffusion system, composed of a donor compartment with diffusional area of 1.54 cm^2^ and a receptor compartment with a maximum volume of 7 mL. As a control, a formulation containing the same components and concentrations used in the VA_DEH was prepared, except by the presence of Poloxamer 407. The experiments were carried out following the methodology described previously by our group [34].

Pig ear skin was chosen for experiments because it presents a close similarity to human skin [35,36]. The pig ears were obtained from a local slaughterhouse and kept under refrigeration for transportation to the laboratory where they were cleaned with water and the skin was separated using a scalpel. The subcutaneous tissue—hypodermis and blood receptor medium—was PBS pH 7.4 with 2% tween^®^ 20. Experiments were carried out at 37 °C under constant stirring (500 rpm) for 24 h. 300 mg of both samples were applied onto the skin of each cell. One-milliliter samples of the receptor medium were collected after 1, 2, 4, 8, and 24 h and replaced with the same quantity of receptor medium. The aliquots were filtered through a 0.45 µm pore size filters disc (WhatmanTM, GE Healthcare UK Limited, Solingen, Germany) and analyzed by HPLC-DAD.

After 24 h, the permeation study was finished. Skins were removed from the Franz type diffusion cell and the excessive formulation was removed with cotton dipped in distilled water. In order to evaluate the drug retained in the epidermis and the dermis, these layers were separated using a scalpel to collect the epidermis [37]. Thereafter, the epidermis and the dermis (cut into small pieces) were inserted in Eppendorf tubes containing 1.5 mL of mobile phase (water 0.1% formic acid + acetonitrile 0.1% formic acid − 9:1 *v*/*v*, respectively) for chlorogenic acid extraction. The Eppendorf tubes were shaken for 90 s and centrifuged for 10 min at 6400 rpm. Then, all samples were filtered through a 0.45 µm pore size filters disc (Whatman) and analyzed by HPLC-DAD [37], as described below (Section 2.7.2).

The results were expressed as mean and standard deviation of one experiment done in quadruplicate.

#### 2.7.2. HPLC-DAD Assay

The content of chlorogenic acid from the permeation studies was evaluated by HPLC-DAD. The separation was performed in a reverse phase column (C-18, 250 mm × 4.6 mm × 5.0 µm; Kromasil, Akzo Nobel, Nashville, TN, USA) in an HPLC-equipped with: ultimate 3000 pump LPG; auto sampler WPS-3000 TSL; columns compartment TCC-3000 SD and diode array detector (DAD) (Thermo Fisher Scientific, São Paulo, Brazil). Water-formic acid 0.1% *v*/*v* (A) and acetonitrile-formic acid 0.1% *v*/*v* (B) were used as mobile phases in the gradient mode: (i) 0–20 min, 10% B; (ii) 21–26 min, 100% B; (iii) 27–37 min, 10% B. The flow rate used was 1.0 mL/min and the injected volume of 70 µL. The detection was performed at 325 nm [29,31].

### 2.8. Statistical Analysis

Each experiment was analyzed separately, using GraphPad Prisma 8.0 Software. *p* values < 0.05 were considered statistically significant.

## 3. Results and Discussion

### 3.1. Chemical Profile of Viscum album Dry Extract

#### 3.1.1. Thin Layer Chromatography

TLC plate of the ethanolic dry extract showed orange-yellowish bands, which are typical of flavonoid compounds at 365 nm UV light, revealed by NP/PEG reagent. The dry extract exhibited one fluorescent blue spot with Rf (retention factor) value, similar to the chlorogenic acid standard (Rf 0.60). According to ANSM [29], chlorogenic acid is a chemical marker of VA species and its identification is important to assure the quality of herbal material. The TLC results obtained with VA_DE are in accordance with previous ones described in the literature [15,31,38].

#### 3.1.2. Determination of Total Flavonoid Content

The flavonoid concentration expressed as mg/g after ethanolic extraction and evaporation was 31.38 mg/g as equivalent of rutin. The content of total flavonoids in VA spp. *abietis* was previously analyzed and varied from 3.200 mg/g to 9.955 mg/g, when extracts produced by different solvents and extraction methods were analyzed [39]. Additionally, the flavonoid content in VA berries from different subspecies (ssp *album* and ssp *austriacum*) and host trees varied from 0.270 to 0.428 mg/g [40]. Tahirovic and Basic [41] evaluated the total flavonoids from the leaves and the stems of VA ssp *album* from different host trees, finding a variation range from 2.29 to 5.05 mg/g.

All these results described in the literature emphasize the influence of subspecies, as well as seasons, solvents, and methodology of extraction, in the flavonoid contents of VA. In fact, the analysis of different ethanolic extracts prepared with unripe berries, stems, and leaves harvested in summer from five different VA host trees showed 4.55 mg/g as minimum flavonoid content, and 9.67 mg/g as the maximum value detected in VA ssp *album* growing on *Ulmus carpinifolia* and *Quercus robur*, respectively [15]. However, for VA ssp *abietis* the less value detected in the flavonoid content (5.25 mg/g) [15] in relation to the present results (31.38 mg/g) could be justified by two major reasons: the difference in the harvest season, the soil influences, and the evaporation of ethanolic solvent. The berries of European VA are ripe and white in winter and unripe and green in summer. The chemical composition of the berries influence the different colors and maturation stages [42]. Additionally, although both VA growing on *Abies alba* have been harvested in Switzerland, the mistletoe samples were collected in different GPS locations, reinforcing the environment influences in VA phytochemical profile, as previously detected by our group [14].

Despite the experimental differences described, *Abies alba* dry extract showed a similar average amount of total flavonoid content when compared to VA var. *coloratum* [43]. Consequently, the methodology applied in the present work could be suggested as a quality parameter for the VA ethanolic and dry extracts.

#### 3.1.3. HPLC-DAD-MS/MS Analysis

**Figure 1:**
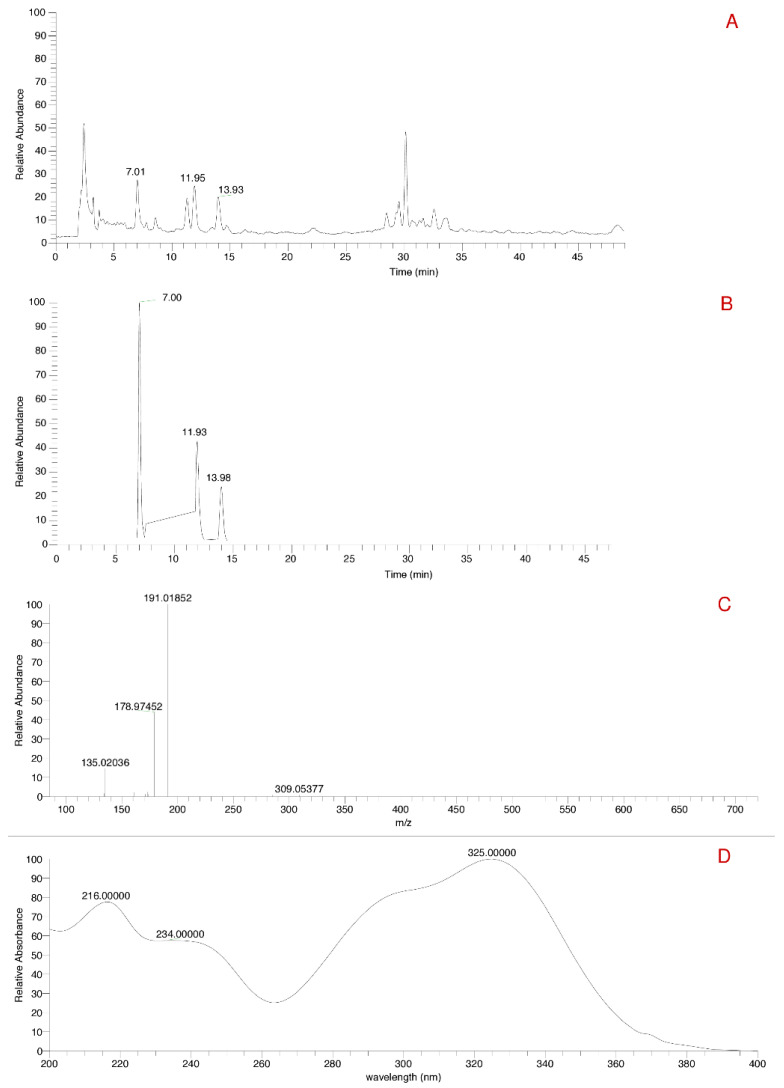
Chromatograms of *V. album* ethanolic dry extract. (**A**)—total ion-chromatogram in negative mode. (**B**)—extracted total ion-chromatogram of chlorogenic acid in negative mode. (**C**)—MS/MS spectra of chlorogenic acid. (**D**)—absorption spectrum in the ultraviolet region of chlorogenic acid.

The VA_DE showed a mass ion of the chlorogenic acid *m*/*z* 353 (Figure 1A). Three peaks were identified for the mass of 353 (Figure 1B). Chlorogenic acid was subjected to fragmentation and the MS/MS spectra is shown in Figure 1C. Figure 1D shows the UV-Vis absorption spectra of chlorogenic acid in the spectral range of 200–400 nm. These peaks at 7.01, 11.93, and 13.98 min suggest the presence of more than one isomer of chlorogenic acid. Mocan et al. [44] described the first peak as 3-*O*-caffeoylquinic acid, the second as 5-*O*-caffeoylquinic acid, and the third as 4-*O*-caffeoylquinic acid. Similarly, identification of different isomers of chlorogenic acid with the same elution chromatographic peaks were previously demonstrated [45], also in VA_DE [31].

Although the biosynthesis of chlorogenic acids is still unclear, except for the 5-*O*-caffeoylquinic acid, the isomerases might be involved with the migration from the main 5- position to positions 3- or 4- [38,46,47].

#### 3.1.4. HPLC-UV-Vis Analysis

**Figure 2:**
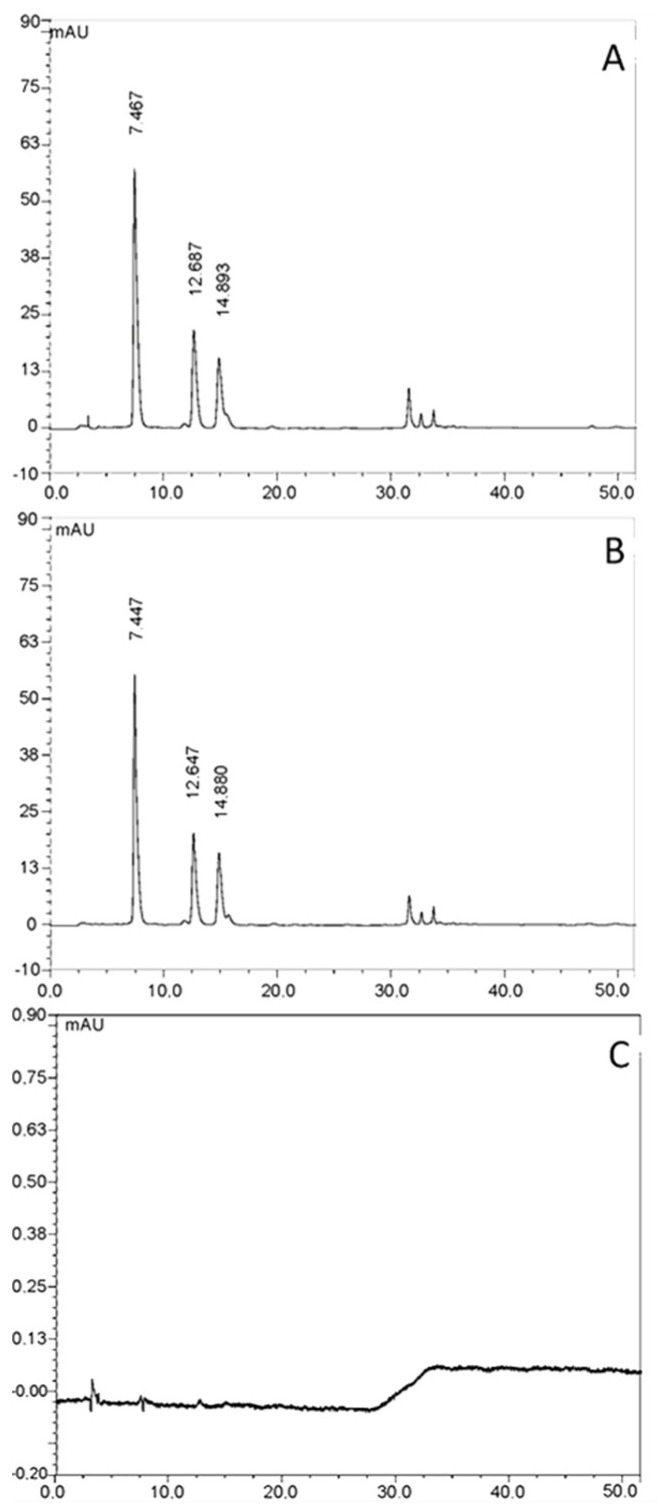
HPLC-UV chromatogram at 325 nm of the (**A**) *Viscum album* dry extract (VA_DE), (**B**) *Viscum album* ethanolic dry extract hydrogel (VA_DEH), and (**C**) vector control (VC, hydrogel without extract). Peaks 1, 2, and 3 correspond to 3-*O*-caffeoylquinic acid, 4-*O*-caffeoylquinic acid, and 5-*O*-caffeoylquinic acid, respectively.

It was possible to identify the chlorogenic acid at the retention time of 7.4, 12.7, and 14.9 min in VA_DE (Figure 2A). The amount of chlorogenic acid was calculated as a sum of the three peaks, resulting in 18.88 mg/g. The chemical profile of VA_DEH and its chemical markers in topical formulations were similar, with the chlorogenic acid isomers at the retention time of 7.4, 12.6, and 14.9 min (Figure 2B), and the same pattern for the VA_DE. This result demonstrated no influence of the hydrogel composition (vector control, Figure 2C) in the HPLC analysis.

Luczkiewicz et al. (2001) [38] quantified the total amount of chlorogenic acid in different subspecies of VA (host trees *Acer platanoides* L. and *Sorbus aucuparia* L.) after exhaustive extraction with methanol solvent. The chlorogenic acid amounts were similar to those detected in the present study after ethanolic extraction of *V. album* ssp *abietis* (host tree *Abies alba*). Our data highlights the high content of chlorogenic acids (18.88 mg/g) detected in the dry ethanolic *Viscum album* extract, when compared to other herbal sources, such as *Coffee canephora*, reported as an important source of chlorogenic acids, ranging from 4.0 to 11.0 mg/g [45].

However, it is important to emphasize the period of the year used in this harvest (winter) and the subspecies analyzed (*abietis*). Urech and Baumgartner (2015) [9] showed important differences in the content of lectins and viscotoxins in VA subspecies when different months of the year were compared. Chemical standardization of a herbal product is important for quality control and to assure its biological activities. The constant quality of the herbal material and also in-process-controls during the production procedure in each and every step leads to the desired batch-to-batch consistency.

### 3.2. Formulation Development

Development of formulations followed the method of the polymer hydration and solubilization. Poloxamer 407 is a copolymer which has the ability to form hydrogels with low polymer concentration in water, around 20% *w*/*w* [24]. Propylene glycol and transcutol^®^ were added to the formulation due to their known ability to enhance transdermal flux of drugs [48,49,50].

Based on visual aspects, VA_DEH shows a brown greenish color due to the VA_DE solubilization, and VA_AEH shows a transparent yellowish appearance. Both hydrogel formulations were submitted to stability, rheology, and in vitro cytotoxicity assays.

### 3.3. Rheology

Rheological analyses were performed due to its importance in the development and the stability parameters related to semisolid formulations. They are considered as non-Newtonian fluids, in other words, complex systems with viscoelastic characteristics [51,52,53].

**Figure 3:**
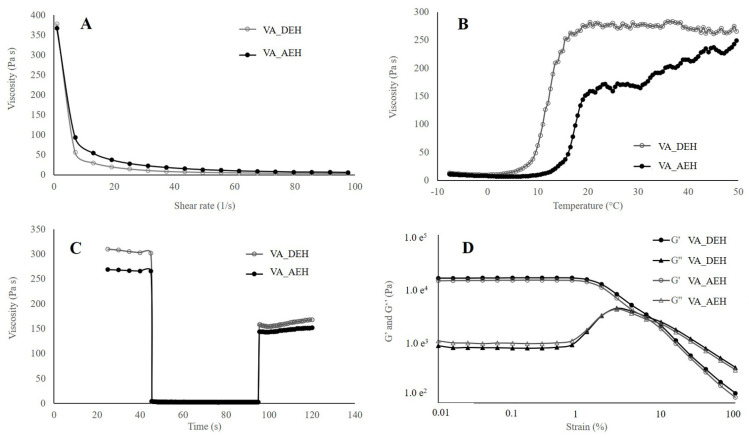
Rheological parameters of the hydrogel containing 5% *w*/*w* of *V. album* dry extract (VA_DEH) and aqueous extract (VA_AEH). (**A**) Apparent viscosity; (**B**) Temperature sweep; (**C**) Three-interval thixotropic test; (**D**) Strain sweep (G′—elastic module; G″—viscous module).

The samples’ flow curve presented a non-linear relation, characteristic of non-Newtonian fluids. Correspondent viscosity curves are presented in Figure 3A. All samples presented a substantial decrease of viscosity in a small range of shear at a constant temperature of 32 °C. The initial (shear rate of 1 s^−1^) and final (shear rate of 100 s^−1^) viscosity for the VA_AEH were 331.0 ± 2.8 Pa.s and 2.8 ± 0.2 Pa.s, and for the VA_DEH were 372.5 ± 7.8 Pa.s and 3.1 ± 0.3 Pa.s, respectively.

Temperature sweep was carried out to follow the samples’ viscosity properties. It is very well known that temperature influences rheological characteristics of poloxamer 407 gels. In higher temperatures, the viscosity is higher, while in lower temperatures (under refrigeration) the polymer solution behaves as a Newtonian. This is the opposite of most materials, which present higher viscosity in lower temperatures. Figure 3B shows better physical stability for the VA_DEH when compared to the VA_AEH from 20–50 °C, probably related to higher content of water in the VA_AEH which weakness the bond’s strength.

Thixotropy evaluated the structural regeneration (e.g., pressure for releasing the formulation form the package) of both hydrogels, in which the recovery percentage after 10 and 60 s are shown in Figure 3C. Both hydrogels presented recovery up to 50% after 10 and 60 s, with the dry extract hydrogel showing a slightly higher recovery in function of time (Table 2).

The elastic (G′) and viscous (G″) modules in function of shear rate, at a frequency of 10 rad.s^−1^, were plotted in Figure 3D. At low deformation (strain), G′ (storage modulus) and G″ (loss modulus) are constant because the samples’ structure is not disturbed. This is called linear viscoelastic (LVE) region, in which both modules are parallel. As soon as the modules start to diminish, the structure is disturbed. Initially, G′ value was higher than G″ value, inferring predominance of the elastic effects (solid-like gels) in both evaluated hydrogels [54]. The limit of this elasticity can also be determined with the amplitude screening test, since the relation between such portions define the strength of the internal network of the hydrogel, which affects the long-term stability.

In Table 3, the intersection value of G′ and G″ is indicated, after which the viscous forces start to prevail over the elastic ones. This intersection point would be the shear range in which the breaking of the material structure occurs, making it leak and behave predominantly as a liquid (G′ > G″) [54]. In addition, this study can be employed to determine the hydrogel strength [55], in which the delta tangent (tan δ) is used, where δ = G″/G′, the ratio between the viscous and elastic modulus. The values are obtained as inversely proportional to the hydrogel strength.

The loss tangent or the internal friction or softening (tan δ) is the ratio between the lost energy by cycle and the stored energy during the cycle. This relation is very useful in the characterization of the hydrogel strength. According to Douglas (2018) [56], fully developed or strong gel has G′ > G″, with more rigid materials presenting smaller tan δ values and more flexible materials presenting higher tan δ values. The hydrogels presented viscoelastic behavior with tan δ values lower than 1, which indicates an existing predominance of elastic gel property [57]. The gelation behavior can be explained as a desolvation and swelling process of the copolymer to form cross-linked aggregates when the temperature is raised and the hydrogen bonds are broken, favoring the hydrophobic interactions of the polypropylene oxide (PPO) domains, defined as a spontaneous micellization process [24,58]. Such behavior was verified in the studied hydrogels with tan δ lower than 1, in which VA_DEH was the strongest one.

A similar profile was previously registered, in which the addition of components such as gellan gum and tween^®^ 80 also increased the gels viscosity [59,60]. Gioffredi et al. (2016) [61] observed the viscosity of poloxamer 407 20% *w*/*v* in PBS (244 Pa.s) and DMEM (240 Pa.s) as lower than the viscosity found in the hydrogels in the present work, which range between 300 and 400 Pa.s. This higher viscosity could be explained by the presence of other components (propylene glycol and transcutol^®^) in the formulations.

### 3.4. Stability Studies

The stability was evaluated during 180 days of storage, at 40 ± 5 °C and humidity of 75 ± 5% in plastic primary packing. The pH analyses were carried out and presented stable values for both samples (Table 4) with an average of 4.50 and 5.38, respectively, to the VA_AEH and VA_DEH.

For cosmetic and topical formulations, compatibility with the skin is important. Maintenance of the skin pH is also an important factor to preserve the cutaneous barrier and an adequate hydration of the skin [62]. The acceptable pH range for topic formulations varies from 4.5 to 7.5 [63]. Our results are compatible with the pH variation desirable to topic formulations, and both hydrogels presented promising results with pH stability at the storage conditions evaluated (Table 4).

The viscosity values were evaluated over the duration of the study in the following intervals: 7, 15, 30, 60, 90, and 180 days. The mean viscosity for the hydrogel with dry extract and aqueous extract was 360.7 and 316.3 Pa.s, respectively, which are statistically different (Welch’s *t* test; *p* < 0.01). Different studies have shown the influence of temperature, poloxamer concentration and the presence of other pharmaceutical components in the formulation viscosity [58,64,65]. Gioffredi et al. (2016) [61] registered lower viscosity values, 244 and 240 Pa.s, when Poloxamer 407, at the same concentration (20% *w*/*v*), was diluted in phosphate buffer saline (PBS) and in cell culture medium (DMEM), respectively. On the contrary, increase in Polaxamer viscosity pattern was detected after the addition of gellan gum and tween^®^ 80 [59,60]. Thus, the higher viscosity detected in our results could also be explained by the additional components (propylene glycol and transcutol^®^) included in both VA hydrogels. Besides, the VA_AEH low viscosity values are possibly related to the presence of an aqueous extract, whereas the VA_DEH presented highest viscosity values, since it was prepared with a dry extract.

The presence of pathogenic microorganisms always affects the shelf time of pharmaceutical products. It can lead to physicochemical disturbance and alter its sterility [66]. None of the samples evaluated presented *S. aureus*, *P. aeruginosa*, *E. coli*, molds, or yeasts (*data not shown*).

In general, the developed hydrogels presented a very good microbiological stability, which could be explained by the presence of propylene glycol at the concentration of 5.0%, showing a preservative property that is comparable to the parabens [67]. Paraben uses in cosmetic and topical formulations has gained attention, due to hepatic and dermal toxicity, skin irritation and higher cancer risk [68,69,70]. The antimicrobial properties of phenolic compounds [71,72] could be related to the microbiological stability of the developed hydrogels. Both aqueous and ethanolic VA extracts present significant amounts of these substances [13,73], which act as natural antimicrobial compounds, avoiding the use of chemical preservatives, such as parabens.

### 3.5. Cell Viability/Proliferation Assay

**Figure 4:**
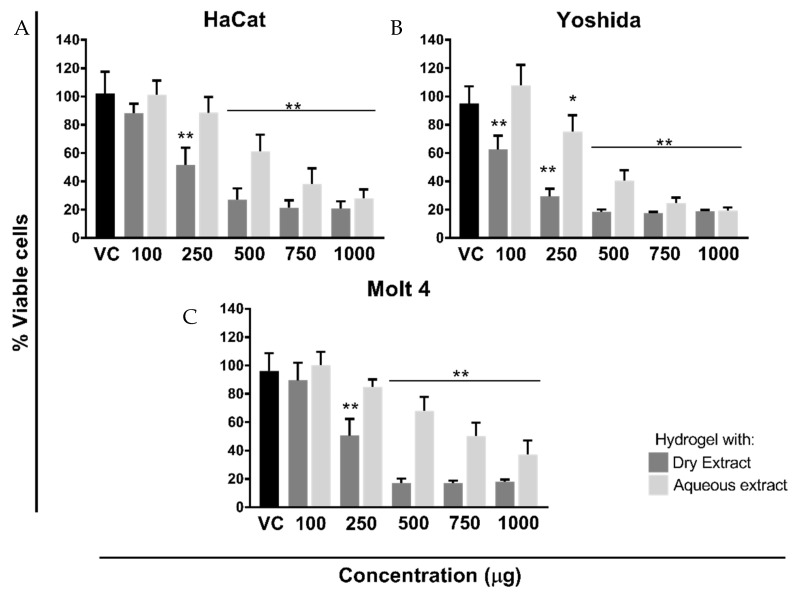
Dose response effect of the hydrogel containing *V. album* dry and aqueous extract on different cell lines proliferation after 24 h of treatment. Cell growth was assessed with WST-1 colorimetric assay, after 1:30 h of incubation. (**A**) HaCat: non-tumoral, adherent; (**B**) Yoshida: tumoral, adherent; (**C**) Molt-4: tumoral, non-adherent. The extract (dry or aqueous) concentration varied between 100, 250, 500, 750 and 1000 μg, corresponding to 2, 5, 10, 15, and 20 mg of hydrogel, respectively. Vector control (VC; hydrogel without extract) was used at a concentration of 20 mg. Results are presented as mean ± SD from three independent experiments in comparison to the VC. * *p* < 0.05; ** *p* < 0.0001, obtained with ordinary one-way ANOVA with Tukey’s multiple comparisons post-test.

The in vitro cytotoxicity/proliferation assay was investigated using two tumoral cell lines (Yoshida, adherent cell line; Molt-4, non-adherent cell line) and one non-tumoral (HaCat, adherent cell line), following WST methodology (Figure 4A–C) [15]. The effects of hydrogel vehicle (vector control; VC) were preliminary evaluated in comparison with untreated cells and the statistical analysis showed absence of cytotoxicity (*p* > 0.05), even at the highest concentration tested (20 mg/mL; data not shown). Therefore, the dose response effect of thermoresponsive hydrogels was compared to the highest concentration of vector control in all cellular experiments (Figure 4).

Since chlorogenic acid is a chemical marker of VA species [29] and was present in VA ethanolic extracts, its theoretical values were calculated following the analytical analysis previously described [15,31]. The same rationality was used in the aqueous extract tested. However, viscotoxin content was used as a reference, following Iscador^®^ specifications (421 µg/mL of total viscotoxin; batch 1804/8141). On Table 5, the respective theoretical values were correlated with the mass (mg) of hydrogel tested on the cellular experiments. These data were used to construct the dose response curves, in which five different concentrations of aqueous and dry ethanolic extract were evaluated. A concentration range from 100 to 1000 µg, corresponding to 2, 5, 10, 15, and 20 mg of VA_DEH and VA_AEH, was evaluated (Table 5; Figure 4).

Both formulations, containing 500 to 1000 µg/mL of VA extracts, significantly decreased the cellular viability with statistical differences (*p* < 0.0001); however, the formulation containing VA_DE presented higher cytotoxicity effect (Figure 4) compared to the VA_AE formulation, which is in accordance with a previous VA cosmetic application study [74].

Although HaCat (Figure 4A) and Molt-4 cells (Figure 4C) exhibited approximately similar sensitivity, the IC_50_ values were subtly different: 5.813 for non-tumor cells; 5.265 mg/mL for tumor Molt-4 cell (Table 6). IC_50_ for the sarcoma cells (Yoshida-adherent) is about half (2.786 mg/mL) of the non-tumoral (HaCat-adherent) cell line. After 24 h of incubation, the percentage of Yoshida viable cells was significantly diminished (*p* < 0.0001), in comparison to the vector control, even at the small VA_DEH concentration (100 µg; Figure 4B). Using 2.5-times higher concentration (250 µg), a reduction of around 60% to 30% on Yoshida viability was significantly detected (*p* < 0.0001; Figure 4B). Similar percentages of viable cells were obtained from 500 µg to 1000 µg, after VA_DEH incubation, with statistically significant differences related to the vector control (*p* < 0.0001). Another biological effect was detected only on Yoshida cells: the VA_AEH at 100 µg/mL slightly increased the cell viability after 24 h (Figure 4B), while the same concentration of VA_DEH induced a statistically significant reduction in the percentage of viable cells (*p* < 0.0001). Further assays should be conducted in order to understand this intriguing experimental result.

In Molt-4 cells, the hydrogel containing 100 µg/mL of VA dry and aqueous extract did not presented statistically significant differences when compared to the vector control (Figure 4C). However, the incubation with 250 µg/mL of VA_DEH significantly diminished (*p* < 0.0001) the cell viability statistically, while 250 µg/mL of VA_AEH was statistically not different from the control.

Previous studies done with VA ethanolic extracts highlight their antitumor potential and showed differences on cellular sensitivity [15,31]. These results were obtained with tumoral cells (B16F10—melanoma murino adherent cells; K562 and Molt-4—human leukemic non-adherent cells; Yoshida—sarcoma adherent cell line), and non-tumoral cells (MA-104—monkey kidney adherent cells; NIH/3T3—mouse embryonic fibroblasts adherent cells). Besides the dose dependent response, our previous study showed the influence of the host trees in the cytotoxic effect. VA ssp *abietis* was the most effective at inducing tumoral damage, even with the smallest ethanolic extract concentration tested [15]. The cytotoxic mechanisms of action triggered by the VA ssp. *abietis* are related to the presence of phenolic acids, flavonoids, lignans, and the high content of viscotoxin A3, inducing mitochondrial damage and cell cycle arrest, as previously described by our group [15,31].

It is interesting to hallmark the reduced sensitivity of normal cells (MA-104 and NIH/3T3) compared to the tumoral cells with regard to VA ethanolic extracts [15,31]. In the present work, this difference was highly evident when HaCat cells were incubated with VA_DEH. This result should be explored deeper in order to confirm this preferential damage detected on tumoral cells instead of non-tumor cells.

### 3.6. Permeation Studies

**Figure 5:**
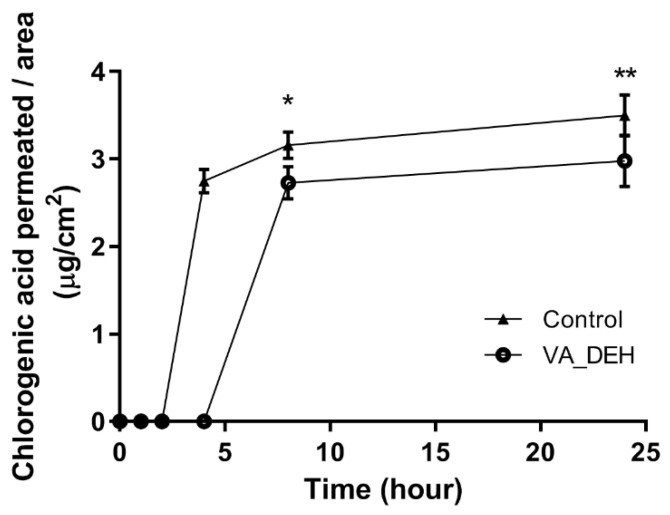
Hydrogel with 5% *w*/*w* dry extract (VA_DEH) permeation assay (mean ± SD). Control was used as the same components of the hydrogel without the polymer. Ordinary two-way ANOVA with Bonferroni’s multiple comparisons post test. * *p* < 0.05; ** *p* < 0.01.

Due to the results of cell viability/proliferation assays, the permeation studies were conducted with the VA_DEH and were compared with control formulation (without Poloxamer 407). Within 4 h of dissolution assay, the chlorogenic acid biomarker permeation was detected throughout the skin from 4 h (Control) to 24 h (Control and VA_DEH), as described in Figure 5.

The amount of chlorogenic acid permeated from the VA_DEH was higher than 2 μg/mL after 8 h, reaching almost 3 µg/cm^2^ after 24 h, which was similar to the control sample already permeated after 4 h of release (Table 7). The lower flow of chlorogenic acid permeated from the thermoresponsive hydrogel is probably related to the presence of poloxamer 407, since its viscosity retards the diffusional release of the drug [20].

**Figure 6:**
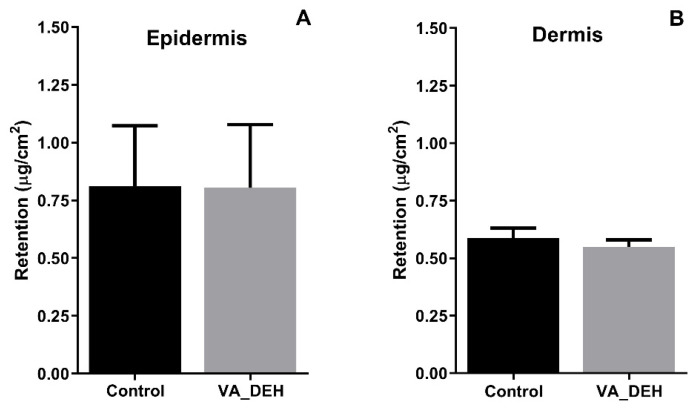
Amount of chlorogenic acid retained in (**A**) epidermis and (**B**) dermis after 24 h of in vitro permeation and retention study (mean ± SD). Welch’s *t* test showed no statistical difference.

In addition to the amount of chlorogenic acid permeated, the amount retained in the epidermis and dermis after 24 h of the experiment was evaluated. The VA_DEH showed some amount of chlorogenic acid being retained both in the epidermis and dermis (Figure 6). However, the epidermis presented more chlorogenic acid than the dermis, which could be explained by the *stratum corneum* layer present in the epidermis, which is a major barrier to drug permeation [36,75]. It could be observed that there was no difference between hydrogel and control, both presenting retention higher than 0.75 μg/cm^2^ in the epidermis and higher than 0.50 μg/cm^2^ in the dermis (Figure 6).

The amount of permeated chlorogenic acid was higher than the one retained due to the presence of components of the studied hydrogel. Besides, the amount of chlorogenic acid permeated through the skin (2.03%—approximately 4.60 µg) is higher than the amount of chlorogenic acid needed to decrease the in vitro cell viability (2 mg VA_DEH—100 µg DE—1.88 µg chlorogenic acid; Figure 4 and Table 5), which emphasizes the antitumoral potential of VA_DEH.

It is already well known that the combination of permeation enhancers improves the drug permeability throughout the skin [76]. The use of transcutol^®^ increases hydrogel permeation by absorbing water due to its hygroscopic nature. Additionally, the propylene glycol humectant properties improve the moisture of the skin and consequently the transdermal potential of the formulation [77,78]. Both permeation enhancers (propylene glycol and transcutol^®^) were used in the developed hydrogels and are probably responsible by the in vitro chlorogenic acid permeation detected (Figure 5 and Figure 6).

These in vitro results highlight the potential transdermal use of VA_DEH as a novel therapeutic approach. Additionally, the dermal compatible pH, the microbiological stability, permeation, and retention properties were never-before-described in thermoresponsive hydrogel containing VA extracts. These interesting pharmaceutical parameters added to the well-known antitumoral potential of natural products, open new possibilities for the VA use transdermally.

## 4. Conclusions

In the present work, an innovative thermoresponsive hydrogel was developed using Poloxamer 407, skin promoters, and *Viscum album* extracts (VA_AE-aqueous and VA_DE-ethanolic dry extract). The addition of skin promoters to the formulation conferred transdermal properties to VA_DEH, which was evaluated by ex vivo skin permeation using chlorogenic acid as a chemical marker. Since the thermoresponsive hydrogel intends to be used as a topical formulation, in addition to the promising antitumoral potential of *V. album* ethanolic dry extract, the cytotoxic effects were evaluated in normal keratinocytes (HaCat) and in tumoral (Yoshida and Molt 4) cell lines. The IC_50_ values showed different cellular selectivity and VA_DEH presented a promising antitumoral potential, even at the smallest concentration tested. The formulation characteristics, such as dermal pH compatibility, microbiological stability, and the non-Newtonian rheological behavior, provide a convenient topic application, manufactured by a simple and easy methodology. All these aspects should stimulate new therapeutic applications for the developed *V. album* hydrogel.

## 5. Patents

The developed formulation was submitted in Brazil (Instituto Nacional de Propriedade Intelectual) requiring intellectual properties, under the number BR 10 2020 003600 9, on 20 February 2020.

## Figures and Tables

**Table 1:** *Viscum album* hydrogels composition.

Formulation	Poloxamer 407	Propylene Glycol	Transcutol^®^	*Viscum album*		Water
				Aqueous Extract (AE)	Dry Extract (DE)	
VA_DEH	20% (*w*/*w*)	5% (*w*/*w*)	5% (*w*/*w*)	-	5% (*w*/*w*) ^a^	q.s. ^c^
VA_AEH	20% (*w*/*w*)	5% (*w*/*w*)	5% (*w*/*w*)	5% (*w*/*w*) ^b^	-	q.s. ^c^

*Viscum album* extracts from the host tree *Abies alba.* % *w*/*w*; VA_DEH (*Viscum album* dry ethanolic extract hydrogel); VA_AEH (*Viscum album* aqueous extract hydrogel); q.s. *quantum satis*. ^a^ Dry ethanolic extract (DE) theoretical chemical marker concentration: 18.88 mg chlorogenic acid/g DE. ^b^ Aqueous extract (AE) theoretical chemical marker concentration 421 µg viscotoxin/mL AE. ^c^ quantity sufficient to 100%.

**Table 2:** Flow recovery of three-interval thixotropic test with different shear rate.

Formulation	Recovery %	
	After 10 s	After 60 s
VA_AEH	54.9%	56.1%
VA_DEH	52.3%	55.3%

**Table 3:** Hydrogel cross over point and tan δ.

Formulation	Damping Factor (tan δ) ^a^	G′ = G″ (γ ^b^, τ_cr_^c^ e G′)
VA_AEH	8.47 e^−2^	γ = 6.79% τ_cr_ = 2.46 e^2^ Pa G′= 2.56 e^3^ Pa
VA_DEH	6.23 e^−2^	γ = 6.75% τ_cr_ = 2.72 e^2^ Pa G′= 2.85 e^3^ Pa

^a^ Calculated between the ratio G″/G′ at deformation of 0.1%. ^b^ γ corresponds to the deformation value at the *crossover* (gel-sol transition point). ^c^ τ_cr_ corresponds to the shear stress at the *crossover* (gel-sol transition point).

**Table 4:** Storage pH and viscosity parameters of *Viscum album* hydrogels. Temperature (40 ± 5 °C), humidity (75 ± 5%).

Storage (Days)	pH		Viscosity (Pa.s)	
	VA_DEH	VA_AEH	VA_DEH	VA_AEH
7	5.48 ± 0.03	4.52 ± 0.02	372.50 ± 7.78	331.00 ± 2.83
15	5.49 ± 0.05	4.51 ± 0.03	368.00 ± 9.89	322.00 ± 5.66
30	5.46 ± 0.04	4.66 ± 0.01	355.50 ± 0.71	328.50 ± 3.53
60	5.24 ± 0.02	4.32 ± 0.00	381.50 ± 10.61	309.50 ± 2.12
90	5.32 ± 0.03	4.55 ± 0.02	370.50 ± 2.12	300.00 ± 5.66
180	5.29 ± 0.03	4.44 ± 0.01	316.00 ± 4.24	307.00 ± 2.83

VA_DEH: *Viscum album* ethanolic dry extract hydrogel. VA_AEH: *Viscum album* aqueous extract hydrogel.

**Table 5:** Corresponding mass of hydrogel, extract, and chemical markers used in cytotoxicity assays.

	*Viscum album* ssp. *abietis*			
Hydrogel (mg)	Dry Extract (µg)	Chlorogenic Acid (µg) *	Aqueous Extract (µg)	Viscotoxin (µg) *
20	1000	18.88	1000	421.00
15	750	14.10	750	315.75
10	500	9.40	500	210.50
5	250	4.70	250	105.25
2	100	1.88	100	42.10

* Theoretical values based in analytical evaluation of each extract.

**Table 6:** Hydrogels IC_50_ values in the tested cell lines.

	IC_50_ (mg/mL)		
Formulation	HaCat	Yoshida	Molt-4
VA_DEH	5.813	2.786	5.265
VA_AEH	12.40	8.856	15.04

**Table 7:** Amount of chlorogenic acid permeated in ex vivo skin permeation test.

Time (h)	Amount of Chlorogenic Acid Permeated (µg/cm^2^) ± SD	
	Control	VA_DEH
4	2.75 ± 0.13	0
8	3.16 ± 0.15	2.73 ± 0.19
24	3.50 ± 0.23	2.98 ± 0.30
